# Highlights for ESMO 40: celebration review for lifetime achievement awards

**DOI:** 10.1136/esmoopen-2015-000010

**Published:** 2016-02-17

**Authors:** Nagahiro Saijo

**Affiliations:** Chief Executive Officer of Japanese Society of Medical Oncology, 2-1-15 Shiba Park Building 6F Hamamatsu-Cho, Minato-Ku, Tokyo105-0013

**Keywords:** JSMO

## Summary

**Figure 1:**
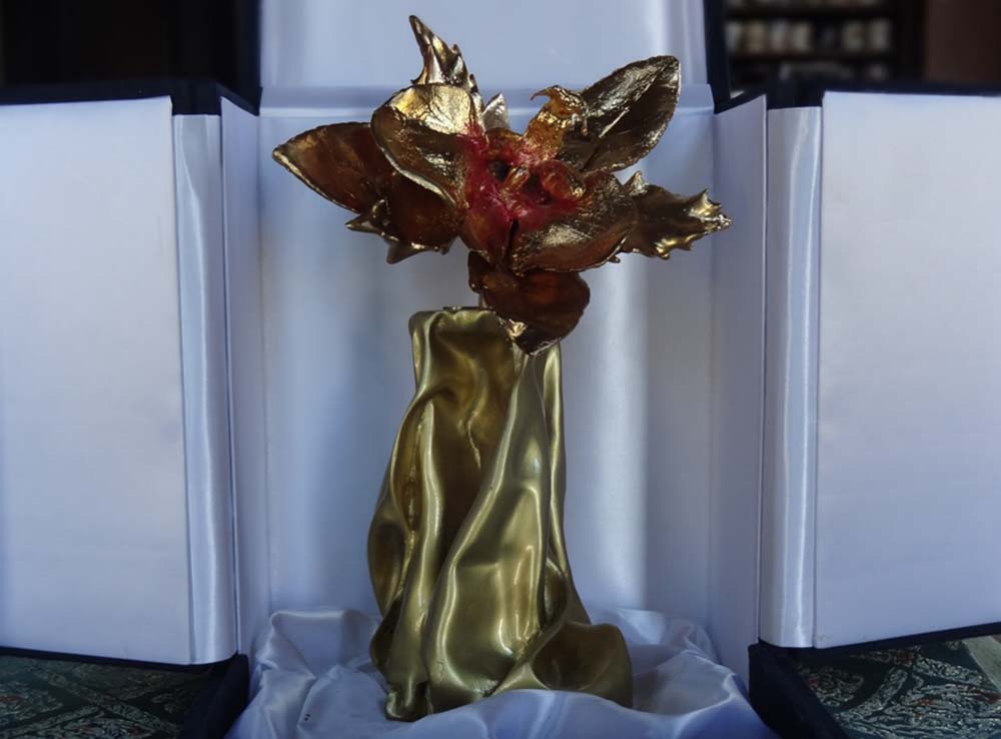
The Flower of Hope—the ESMO Lifetime Achievement Award.

My work, my crucial achievement, has been in the management of the transition of the conduct of cancer research/management from basic researchers/surgeons to medical oncologists (figure 1).

This transition can be divided into four categories:

1. The development, progression and consolidation of global Medical Oncology in Asian countries, especially in Japan.
2. Building a close collaboration with European Society of Medical Oncology (ESMO) and receiving strong support from ESMO, through (A) Adaptation of the ESMO/ASCO Core Curriculum in Medical Oncology,^1^ (B) Mutual ESMO/*Japanese Society of Medical Oncology* (JSMO) joint symposia during their respective meetings, (C) making Annals of Oncology JSMO's official scientific publication,^2^ along with (D) my own work as an Associate Editor of Annals of Oncology (2011–2014), (E) my work as ESMO's National/Regional Representative and Membership Committee member (2005–2011).
3. Leadership of clinical research in Japan as a key opinion leader and primary investigator in IND (investigational new drug) trials and a chairman of the *Japanese Clinical Oncology Group* (JCOG; 2000–2009), organising a governmental clinical trial group; and the development of new gold standards in the treatment of malignant diseases, especially in thoracic malignancy by investigator-initiated randomised controlled clinical trials.
4. Encouraging translational research and the publication of numerous articles, especially in the area of the identification of molecular targets for anticancer drugs, as Chief of the Pharmacology Division in the National Cancer Center Research Institute.

## Preface

When I began my training in Medical Oncology, cancer research, including translational research, was conducted by basic researchers, and cancer treatment, including chemotherapy, was conducted by surgeons. There was no place for medical oncologists to play an active part in cancer research and treatment. ESMO was established 40 years ago and now has more than 10 000 professionals as members, and medical oncology has been recognised as a subspecialty of Internal Medicine in Western countries. However, there were no such comparable developments in Japan. There had been no systematic education systems for medical oncology in the majority of universities in Japan, and it had only been addressed during training regarding the possible diseases afflicting each organ. For a long time in Japan, cancer was considered as a disease for only surgeons to treat. Recently, many new drugs which require specialised knowledge and skills have been introduced into clinical practice.^3^ In addition, progress in molecular biology and clinical studies has clarified common features in cancers from different organs.^4^ ^5^ It is clear that systemic cancer therapy should be performed by medical oncologists with sufficient knowledge and a level of clinical skill based on cross-specialty training.

## The creation of the JSMO

**Figure 2:**
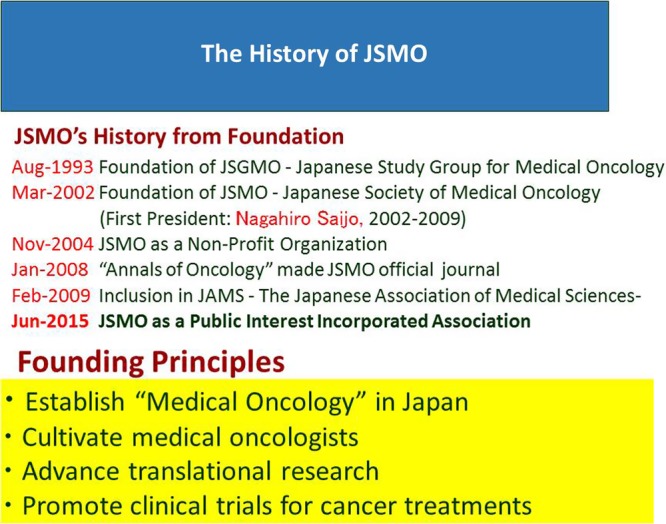
The history of the Japanese Society for Medical Oncology.

**Figure 3:**
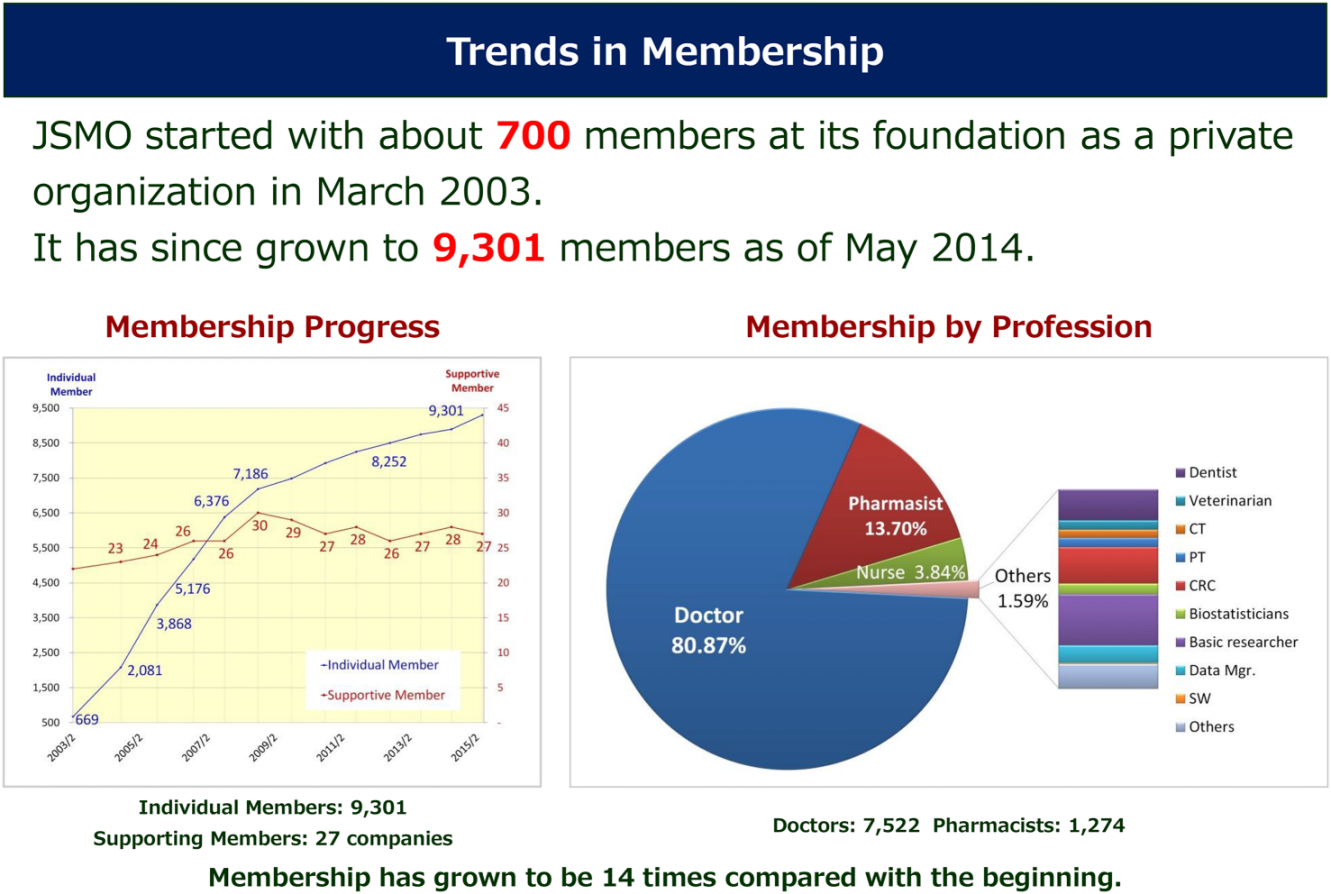
Trends in JSMO growth. JSMO, *Japanese Society of Medical Oncology.*

In 1993, the *Japanese Study Group for Medical Oncology* (JSGMO), the antecedent of the JSMO founded by myself and Dr Masahiro Fukuoka and Dr Yutaka Ariyoshi, started an annual meeting to strengthen the recognition of medical oncology in Japan (figure 2). JSGMO organised a symposium once or twice a year on clinical trials and translational studies, with 16 symposia held until 2002. However, we could not increase the number of members involved in medical oncology, and there was little excitement for clinical trials during that time. The average numbers of attending scientists were 500–800, even though ICH-GCP for IND trials was initiated in 1998. JSGMO's strategic plan was then fundamentally renewed to establish a system for the board certification of medical oncologists in Japan. For this purpose, JSGMO was renewed as JSMO in 2002. At the first meeting, approximately 600 members attended and discussed future perspectives in Medical Oncology. The infrastructure of JSMO was consolidated to enable the realisation of examinations for the board certification of medical oncologists. For example, JSMO provides CME credit for attending educational events. In addition to its annual scientific meeting, JSMO organised educational seminars twice a year for developing essential knowledge of Medical Oncology, and a best of ASCO meeting as an advanced course. At both activities, 500–800 investigators attended. JSMO published two textbooks on medical oncology, one for undergraduate students: *INTRODUCTION OF MEDICAL ONCOLOGY*^6^ and another for young postgraduate doctors who would be candidates to become Board Certified Medical Oncologists: *NEW TEXTBOOK OF MEDICAL ONCOLOGY.*^7^ The number of members rapidly increased and as of November 2015 reached more than 9300, with more than 5500 investigators attending the Annual meeting (figure 3).

From the beginning, global Societies such as ESMO and ASCO have supported JSMO in various ways. For example, JSMO adopted the Global Core Curriculum of ASCO/ESMO,^1^ and recently JSMO adopted ASCO's Conflict of Interest policy.^8^ Annals of Oncology, the official journal of ESMO, also became that of JSMO in 2008. Examinations for the board certification of medical oncologists began in 2005.

**Figure 4:**
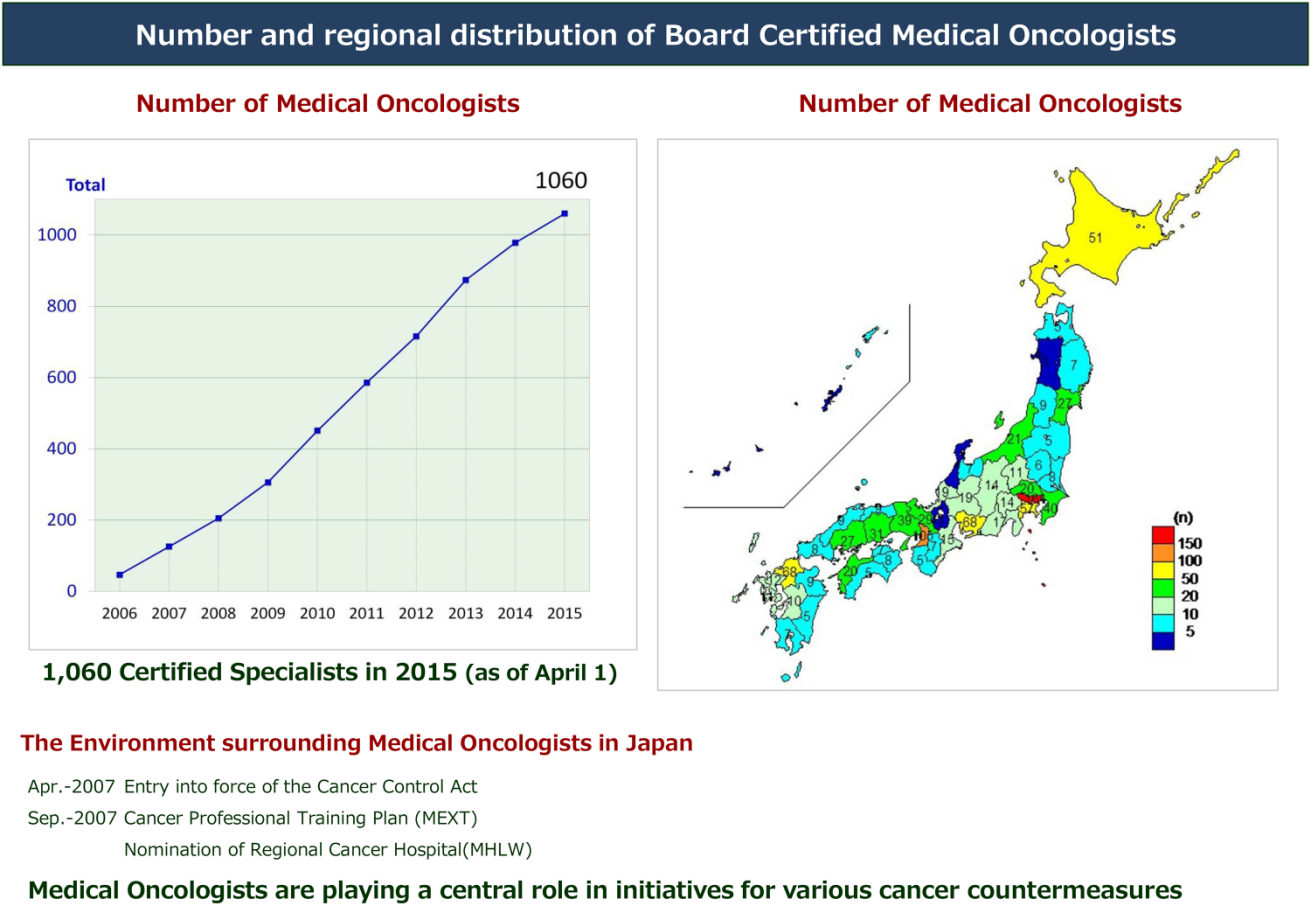
JSMO board Certified Medical Oncologists. JSMO, *Japanese Society of Medical Oncology.*

To apply for the examination, an applicant should have (A) worked for at least 5 years in a JSMO-certified hospital after completing 2 years of basic clinical training and (B) submitted 30 case reports which should include haematological, breast, thoracic and gastrointestinal malignancies. The examination includes both oral and written components. In 2006, 47 individuals passed the first examination session and became Board Certified Medical Oncologists. As of 2015, a total of 1060 physicians have become JSMO Board Certified Medical Oncologists (figure 4). The pass rate for the examination is approximately 60%, which is low compared with that for certified specialties in other societies. However, JSMO views this lower rate of success in a positive light as the Society wants to certify only well-educated and qualified medical oncologists, and therefore JSMO intends to maintain this relatively low pass rate.

## Requirements for a medical oncologist certified by JSMO

Certified Medical Oncologists are specialised physicians trained in the investigation and care of patients with cancer. They play an integral role in cancer management by multidisciplinary teams, providing special expertise to patients through their knowledge of systemic treatment of cancer with cytotoxic chemotherapy, hormone therapy, molecular targeted drugs and immunotherapy. In Japan, these therapies have been provided historically within the framework of medical subspecialties based on the specific organ in which each cancer developed. JSMO certifies physicians who are capable of providing systemic therapy reflecting the progress of cancer research as Board Certified Medical Oncologists.

Board-Certified Medical Oncologists are required to:

1. Understand the biology of cancer and the clinical pharmacology of the drugs used to treat this disease;
2. Develop new treatment strategies through translational research;
3. Learn skills in management and service delivery for various cancer sites and to understand the complex issues, such as clinical conditions and the social background of each patient, involved in introducing new treatments;
4. Have a central role in the planning, conduct and promotion of clinical trials based on scientific methodology and logical ideas;
5. Learn and understand palliative care in order to provide expert consultations;
6. Play a definite role in the practice of treating oncology emergencies, a service designed to ensure appropriate and urgent management of complications of cancer itself and its treatments;
7. Integrate a team of medical staff with multiple specialties;
8. Contribute to the development of medical oncology by educating trainees and to provide an excellent educational environment;
9. Be a driver for the prevention of cancer.

JSMO contributes to the progress of cancer treatment and public health by certifying well-trained physicians as Board Certified Medical Oncologists.

## The contribution of JSMO to the basic act for anticancer measures in Japan

The Japanese government has now also recognised the importance of both medical oncology and radiation oncology. Historically, the majority of cancer care in Japan, including chemotherapy, was performed by surgeons. On 16 June 2006, the Japanese government enacted a law specifically focused on cancer and its management, the ‘*Basic Act for Anticancer Measures*’.

The law came into force on 12 April 2007, some 5 years after the founding of JSMO. The law has three basic policies:

Section 1, promotion of prevention and earlier detection of cancer; Section 2, promotion of full equality of medical care for all cases of cancer; and Section 3, the promotion of cancer research. To realise Section 2, Article 14 states as follows: “The Government and Prefectures shall establish necessary policies to bring up medical doctors and other medical staff who have expertise in surgery, radiotherapy, chemotherapy, and other cancer managements.”

To give shape to the missions and visions of the Act, the Ministry of Health, Labor, and Welfare (MHLW) started to nominate prefectural and regional ‘Cancer Management Hospitals’, known as Chiiki Gann Shinryo Renkei: Kyoten Byouin—Regional Cancer Care Core Hospital-, which should have appropriate medical staff members and facilities.

In addition, the Ministry of Education, Culture, Sports, Science, and Technology (MEXT) has proposed the Cancer Professional Educational Plan, known as GANNPRO. The main purpose of GANNPRO is to increase the number of cancer specialists, including medical and radiation oncologists, oncology pharmacists and nurses, by demonstrating support for these specialties.

The efforts of MHLW and MEXT seem outwardly to be changing medical practice for cancer treatment in Japan. The number of registered Cancer Management Hospitals has increased to approximately 400 and new divisions of medical and radiation oncology have been founded at several universities. However, more than 60% of registered Cancer Management Hospitals still have no JSMO Board Certified Medical Oncologists. All patients have the right to receive reasonable medical care in qualified hospitals and, in order to address this issue, the criteria for the registration of Cancer Management Hospitals by MHLW should be revised. Furthermore, hospitals that have at least two or three JSMO Board Certified Medical Oncologists should be financially rewarded to improve their infrastructure.

## Future directions of JSMO as a global society for oncology

JSMO was initially established as an educational organisation, with practical success in instruction regarding the role of medical oncology in Japan. New information on translational/basic research and clinical trials is primarily reported during either the ASCO or ESMO meeting, not during the JSMO annual meeting. Despite the continued importance of educational activities at the JSMO annual meeting, the focus on scientific data regarding new targeted agents is increasing in Japan, as well as in other Asian countries.^9^ Some molecularly targeted drugs have exhibited substantial differences among ethnicities regarding effectiveness and toxicities.^10^ ^11^ In addition, research in diseases prominent in Asia, such as gastric cancer and hepatocellular malignancies, is also developing.

**Figure 5:**
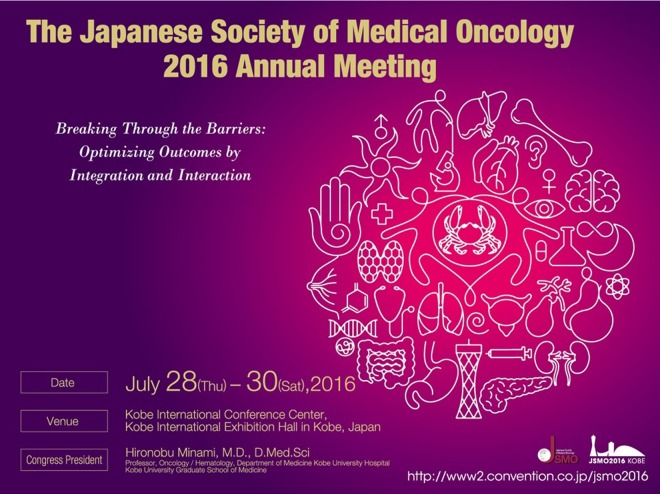
The JSMO 2016 Annual Meeting. JSMO, *Japanese Society of Medical Oncology. Japanese Society of Medical Oncology*.

The next JSMO annual meeting is on 28–30 July 2016 in Kobe, Japan and the Meeting President will be Professor Hironobu Minami (figure 5). Through collaborations with the Chinese Society Clinical Oncology, the Korean Association of Clinical Oncology, Singapore Society of Oncology and the Medical Oncology Group of Australia Incorporated, JSMO will contribute to the promotion of activities in the field of medical oncology in Asia.

## Establishment of global standards for treatment of lung cancer

### Contribution for the development of new anticancer drugs

The development of new anticancer drugs is extremely important to improve the survival time of patients with cancer. From 1989 to 1997, I worked as a chairman of the Pharmacology Division of National Cancer Center Research Institute. Famous pharmacologists were invited on the basis of a Comprehensive 10-year Strategy for Cancer Control to transfer new ideas and techniques. They included Dr Anne W Hamburger, Dr Peter Twentyman, Dr Kenneth D Tew, Dr John Lazo, Dr Youcef Rustum, Dr Enrico Mihich, Dr Eckard Podak, Dr Kristin Olsen, Dr Awtar Krishan, Dr Suzan Arbuck, Dr Rosemarie Mick, Dr Dean Brenner, Dr David Curiel, Dr Haim Tapiero, Dr K Bojanowsky, Dr Wang S Hong, Dr Young S. Lee and Dr Hyo J. Kuh. Many mechanisms of drug resistance, especially for platinum compounds, have been identified,^12–17^ and these studies led to the clarification of molecular targets of cytotoxic drugs. Many drug-resistant cells were provided to pharmaceutical companies to screen better anticancer drugs. The suggestions for best administration schedules and combination regimens have been obtained from these translational studies.^18^ The trial for the selection of a personalised regimen was done by drug sensitivity testing.^19^ In my role as a primary investigator for the efficient and scientific development of a majority of key anticancer drugs, pharmacokinetic/pharmacodynamic studies done by Pharmacology Division were extremely important.^20–24^

### Activity in the JCOG

JCOG, supported by governmental funds, has been initiated as a multidisciplinary treatment group for malignant diseases in 1978^25^ ^26^ and organised as a cooperative study group which has by-laws, a protocol review system and a monitoring committee since 1985. JCOG conducts multidisease, multidisciplinary treatments including commercially available drugs, radiation therapy and surgery. JCOG consists of an Executive Committee, Data Center, Operational Office and 16 Study Groups covering the majority of organ tumours except for paediatric tumours and leukaemia. The Lung Cancer Study Group (LCSG) led by myself joined in 1982 and it played a major role in the establishment of JCOG itself because LCSG is a pioneer and opinion leader for clinical trials, especially randomised controlled trials. I worked as a JCOG chair from 2001 to 2009. I invited Dr Steven Piantadosi and Dr Robert Makuch and worked with them to strengthen the statistical centre.^27^ ^28^ At that time, there was only one staff member in the operational office. Specialists of medical oncology and radiation oncology in the field of lung cancer have been invited on the basis of a 10-year Comprehensive Cancer Control Strategy and we worked with them. They included Dr James R Jett, Dr Bruce E Johnson, Dr Andrew Trissi, Dr David H Johnson, Dr Jack Ruckdeshel, Dr Everett E Vokes, Dr Roy R Herbst, Dr Raymond Ablatt, Dr Wilfred EE Eberhardt, Dr Rafael Rosell and Dr Sumitra Thongprasert. During my term of office as a chairman, the number of staff members in the JCOG data centre has dramatically increased and the study design of clinical trials has been globalised. In addition, other cooperative study groups which have a data centre and operational offices have begun to be organised. In recent years, total protocols handled by JCOG are 70–100, and patient accrual is 2600–3000 per year. In addition to the studies of LCSG, many prestigious clinical trials have been conducted, especially in the comparison of surgical procedures such as “Left thoraco-abdominal approach versus abdominal-trans-hiatal approach for gastric cancer of the cardia or subcardia: a randomised controlled trial” 29 and “D2 Lymphadenectomy alone or with para-aortic nodal dissection for gastric cancer”. 30 Recent major publications of JCOG studies from 2011 to 2015 were in Lancet Oncology (4), J Clin Oncol (5), Ann Surgery (2), Annals Oncol (4), and Br J Surgery (5). Among them, five were studies of LCSG and the cumulative impact factor is highest in LCSG.

### Small cell lung cancer

As part of JCOG (Japanese Clinical Oncology Group), our group has conducted many pivotal trials for the establishment of global standards of care for limited and extensive small cell lung cancer (SCLC).

When I graduated from University, SCLC was primarily treated with mitomycin C alone in the National Cancer Center (Japan). In the late 1970s, ACNU (a water-soluble nitrosourea compound), developed by Sankyo Co (Japan), showed surprising antitumour effects in SCLC preclinically,^31^ and clinically it was very active in extensive disease (ED)-SCLC.^32^ The survival time of ED-SCLC treated with ACNU has reached the state of the art in the 1970s. Unfortunately, further development of the drug after phase II trials was stopped because of prolonged thrombocytopenia. JCOG conducted a three-armed RCT (randomised controlled trial) comparing CAV (cyclophosphamide+adriamycin+vincristine) versus PE (cisplatin+etoposide) versus CAV alt PE to determine new standard chemotherapy of PE and also to evaluate non-cross resistant chemotherapy. CAV alt PE showed superiority only in LD (limit disease)-SCLC by post study stratification. Although the results were essentially negative, the data were published in the Journal of the National Cancer Institute^33^ and investigators of the cooperative study group were encouraged because of the first completion of a large-scale randomised trial in Japan. Adverse events for the PE regimen were mild compared with other regimens, and this regimen has contributed to the establishment of a global consensus in the SCLC workshop held in Helsingor (Denmark). Another key trial against LD-SCLC comparing concurrent versus sequential chemoradiotherapy (accelerated hyperfractionation) could not meet the primary end point (the superiority of the concurrent arm) statistically, although it showed a tendency to better survival in the concurrent group. The results were published in the Journal of Clinical Oncology,^34^ and a global consensus in favour of concurrent chemoradiotherapy in LD-SCLC was obtained. Many strategies to improve the treatment of SCLC failed to show survival benefit for new treatments.^35^ An RCT comparing PE versus IP (irinotecan+cisplatin) conducted by JCOG clearly showed the survival benefit of the IP regimen and the results appeared in the New England Journal of Medicine.^36^ Although American studies failed to demonstrate reproducibility, the IP regimen became one of the standards for the treatment of ED-SCLC. Many trials were performed with newly developed amrubicin in Japan, which has recently been used as a second-line regimen.^37^ ^38^ Recently, JCOG reported that a weekly IPE (irinotecan+cisplatin+etoposide) regimen shows better survival compared with the US standard: topotecan as a second-line treatment after IP or PE failure.^39^

### Non-small cell lung cancer

No active drug existed for the treatment of non-SCLC (NSCLC) before the development of CDDP (cisplatin). Combinations of 3–5 drugs failed to obtain reproducible responses and survival benefit of chemotherapy was viewed sceptically in NSCLC before 1980.

Our first RCT in NSCLC was the comparison of carbazilquinone+MMC versus pepleomycin+MMC. The response rates of both regimens were less than 15% and OS was less than 8 months. Death by interstitial pneumonia was observed in 13.2% of patients (Cancer Treatment Report).^40^ After this, we conducted a phase II study of single agent chemotherapy of MMC, tegaful, ACNU, UFT(uracil+tegaful), VDS(vindesine), VP-16(etoposide), VBL(vinblastine), CDDP, KW-2083(MMC derivative), CBDCA(carboplatin) and 5’-DFUR to search for active drugs for NSCLC. Only CDDP, VDS and MMC showed responses of 15%. The combination of CDDP+VDS had already been reported by Dr Richard Gralla who used 120 mg/m^2^ of CDDP.^41^ ^42^ Since that dose was not tolerable, we conducted a dose-finding study and identified the appropriate dose of the drugs: 80 mg/m^2^ Q3wks and 3 mg/m^2^ weekly for CDDP and VDS, respectively.^43–45^ This regimen survived as a standard until the third generation of drugs was introduced. CPT-11 was the first drug used for comparison. An RCT of CPT-11+CDDP versus VDS+CDDP demonstrated a favourable response and survival with the CPT-11 containing regimen, although there was no statistical difference.^46^ Using CPT-11+CDDP as a reference regimen, we conducted a four-arm RCT named the FACS (Four Arm Cooperative Study) trial with VNL (vinorelbine)+CDDP, GEM (gemcitabine)+CDDP and PTL(paclitaxel)+CBDCA (carboplatin) to demonstrate non-inferiority of regimens containing third generation drugs.^47^ Another RCT comparing VDS+CDDP versus DTX (Docetaxel)+CDDP showed the superiority of DTX+CDDP.^48^ These trials contributed to the consensus that platinum-doublet chemotherapy including a third generation drug is a standard for the treatment of NSCLC.^49^ ^50^

The average age of patients with lung cancer is reaching 75 years. In the majority of them, full-dose chemotherapy cannot be given because of the high frequency and grade of haematological toxicities and comorbidities. JCOG has tried to develop the most appropriate regimen for this population.^51^ ^52^ However, after the appearance of Epidermal Growth Factor Receptor-Tyrosine Kinase Inhibitor (EGFR-TKI), it became very difficult to analyse the survival, if the trial included of EGFR wild-type and mutation-positive patients. Within JCOG, docetaxel alone is still a standard regimen in elderly patients with advanced NSCLC. In stage III locally advanced elderly patients, JCOG has demonstrated that the addition of daily low-dose carboplatin improves survival.^53^

Gefitinib was approved in Japan in 2002, with highly responsive patients observed during phase 1 and 2 trials. The Asian Global Trial, IPASS, showed that Gefitinib is active only in EGFR-Mt+patients.^54^ Many trials on EGFR-Mt+patients completely changed the concept of chemotherapy in advanced NSCLC, and median survival of this population is now reaching 35–40 months by means of the combination of cytotoxic chemotherapy and EGFR-TKIs. It became quite difficult to compare the survival before or after the appearance of EGFR-TKIs. In addition, treatment of NSCLC has recently been discussed on the basis of histological subtypes such as squamous and non-squamous. There is no comparability for survival times of all comers and selected populations of NSCLC.^55^

Introduction of 3rd generation TKIs and PD-1 antibodies will further improve the outcome for patients with NSCLC.^56^ ^57^ Non-SQ (non-squamous cell carcinoma) has been classified into various genetic groups and studies based on precision medicine for each group are underway. The Japanese contribution in this area can be expected to provide magnificent treatment tools for NSCLC.
